# The activity of *Levisticum officinale* W.D.J. Koch essential oil against multidrug-resistant *Mycobacterium tuberclosis*

**Published:** 2018-12

**Authors:** Mansour Miran, Mohammad Mehdi Feizabadi, Hossein Kazemian, Jalil Kardan-Yamchi, Hamid Reza Monsef-Esfahani, Samad Nejad Ebrahimi

**Affiliations:** 1 Department of Pharmacognosy and Biotechnology, School of Pharmacy, Ardabil University of Medical Sciences, Ardabil, Iran; 2 Department of Microbiology, School of Medicine, Tehran University of Medical Sciences, Tehran, Iran; 3 Thoracis Research Center, Imam Khomeini Hospital Complex, Tehran University of Medical Sciences, Tehran, Iran; 4 Department of Pathobiology, Division of Microbiology, School of Public Health, Tehran University of Medical Sciences, Tehran, Iran; 5 Department of Pharmacognosy, School of Pharmacy, Tehran University of Medical Sciences, Tehran, Iran; 6 Department of Phytochemistry, Medicinal Plants and Drugs Research Institute, Shahid Beheshti University, Tehran, Iran

**Keywords:** *Levisticum officinale*, Multidrug-resistant-*Mycobacterium tuberculosis*, Essential oil, Molecular modeling

## Abstract

**Background and Objectives:**

Essential oils are used for controlling and preventing human diseases and the application of those can often be quite safe and effective with no side effect. The essential oils have been found to have antiparasitic, antifungal, antiviral, antioxidant and especially antibacterial activity including antibacterial activity against tuberculosis. In this study the chemical composition and anti-TB activity of essential oil extracted from *Levisticum officinale* has been evaluated.

**Materials and Methods:**

The essential oil of *L. officinale* was obtained by the hydro distillation method and the oil was analyzed by GC-FID and GC-MS techniques. The antibacterial activity of essential oil was evaluated through Minimum Inhibitory Concentration (MIC) assay using micro broth dilution method against multidrug-resistant *Maycobacterium tuberculosis*. The molecular modeling of major compounds was evaluated through molecular docking using Auto Dock Vina against-2-trans-enoyl-ACP reductase (InhA) as key enzyme in *M. tuberclosis* cell wall biosynthesis.

**Results:**

The hydrodistillation on aerial parts of *L. officinale* yielded 2.5% v/w of essential oil. The major compounds of essential oil were identified as α-terpinenyl acetate (52.85%), β- phellandrene (10.26%) and neocnidilide (10.12%). The essential oil showed relatively good anti-MDR *M. tuberculosis* with MIC = 252 μg/ml. The results of Molecular Docking showed that affinity of major compounds was comparable to isoniazid.

**Conclusion:**

The essential oil of aerial parts extracted from *L. officinale* was relatively active against MDR *M. tuberculosis*, and molecular docking showed the major compounds had high affinity to inhibit 2-trans-enoyl-acyl carrier protein reductase (InhA) as an important enzyme in *M. tuberculosis* cell wall biosynthesis.

## INTRODUCTION

Essential oils are beneficial for controlling and preventing human diseases and the application of those can often be quite safe and effective with no side effect. The essential oils have been found to have antifungal, antiparasitic, antiviral, antioxidant and especially antibacterial activity (1). The essential oil can be used for the treatment of different diseases including atherosclerosis, thrombosis and diabetic (2). Also the essential oils play an important role regarding inhibiting growth of *M. tuberculosis* and it can be mentioned that essential oils of *Salvia aratocensis* (Beijing genotype strains), *Eugenia caryophyllata, Cuminum cyminum,* and *Cinnamomum verum* (strain H37Rv) also have this feature as well (3–4).

*Levisticum officinale* belongs to Apiaceae family and it grows in the Hezar Mountain located in Kerman province, Iran (5). *L. officinale* as a medicinal plant is used in the treatment of urinary tract infection and kidney stone (6). Its oil is used in medicinal preparation, food flavoring and aromatherapy (7).

Although the antibacterial activity of *L. officinale* extract has been evaluated on some *Mycobacterium* species (8–9), there is no report about anti- *Mycobacterium* activity of essential oil extracted from this plant. So in this study, we aimed at evaluating the antibacterial activity of *L. officinale* essential oil against MDR *M. tuberculosis* strain.

## MATERIALS AND METHODS

### Plant material.

The aerial parts of *L. officinale* were collected in 2016, from the Hezar Mountain located in Kerman province, Iran. The plant material was identified by Prof. Farideh Attar. A voucher specimen (46553-TUH) has been deposited in the herbarium of Science Faculty of Tehran University.

### Isolation of essential oil.

The aerial parts (150 g) of *L. officinale* was crushed and then the essential oil was isolated by hydro distillation using a Clevenger type instrument for 3 hours. Finally, the oil was dried with anhydrous sodium sulfate and stored in dark vials at −20°C before analysis.

### Analysis of the essential oil: GC-MS analysis.

Thermoquest Finnigan Trace GC–MS instrument equipped with a DB-5 column (30 m × 0.25 mm; 0.25 μm film thickness) was used for GC-MS analysis. The temperature of the oven was set from 60 to 250°C at 4°C/min, and then isothermal for 15 min. The FID and injector temperatures were programmed at 240°C and 250°C, respectively. Helium was used as carrier gas at the constant flow rate of 1.1 ml/min and a split ratio of 1:100. Mass range was 45–450 amu and the MS operating parameters were: ion source temperature, 200°C; ionization voltage, 70 eV (7).

### GC-FID analysis.

The Agilent gas chromatograph (FID) with a DB-5 fused silica column (30 m × 0.32 mm; 0.25 μm film thickness) was used to GC analysis. Nitrogen was used as a gas carrier at a constant flow of 1.1 mL/min, a split ratio of 1:50. The oven temperature was set from 60 to 250°C at 4°C/min, and then isothermal for 15 min. The injector and FID temperatures were programmed at 240°C and 250°C, respectively.

### Identification of components.

The constituents of the volatile oil were recognized by the calculation of their retention indices under temperature-programmed conditions for n-alkanes (C_6_–C_24_) and the oil on DB-5 column under the same conditions. Identification of individual compounds was completed by the mass library, comparison of their mass spectra with the MS literature data (10–11) and retention indices (RI). GC-FID peak area was used to calculate of the percentage compositions without the use of the correction factor.

### Molecular docking studies.

The structure of compounds (1–4 and isoniazid), were drawn by Chem Draw Professional 15.0, and the structures were transferred to Chem3D and were saved as PDB format. Then in order to perform the energy minimization of the structures MM2 force field method was used. Finally minimized ligands in the PDB format were saved as PDBQT files using the Auto-Duck tool. The crystal structure of InhA with PDB ID: 1BVR was obtained from the RCSB Protein Data Bank (PDB). The protein structure was prepared by removing water and adding hydrogens and the protein file was saved as the PDBQT format for molecular docking studies.

Molecular docking was performed for determining binding modes of the ligands using Auto-Dock Vina software (12). The binding site residues of InhA with grid center at X:12.832, Y:16.388, Z: 6.306 and the number of points in each dimension as X:20, Y:20, Z:20 and Spacing (Å): 1.0 were originated from a previous study (13). Binding site residues and grid box of InhA were shown in Fig. 2. In order to show interactions between ligand and receptor Discovery Studio Visualizer v4.5 was used.

### Bacterial strains.

An isolate of MDR *M. tuberculosis* was obtained from the microbial collection of Department of Medical Microbiology, Tehran University of Medical Sciences. The antimicrobial susceptibility testing was done according to CDC standard proportional method for multi-drug resistance confirmation of *M. tuberculosis* (14–15). *M. tuberculosis* H37Rv was used as standard strain.

### Determination of MIC.

The serial dilution of each essential oil was completed in a concentration ranged from 0.250 to 1008 μg/mL in the sterile 96 wells. Also, Mueller-Hinton broth medium supplemented by 0.5% tween 80 was used as co-solvent as well as trace value. The suspensions were diluted in seven serial dilutions. The 100 μl of solution was transferred from column 1 to next column, and identical serial 1:2 dilutions were continued through column 7 (16). Each well was inoculated with 5μl of 0.5 McFarland standard turbidity of bacterial suspensions (17). A column without essential oil was inoculated as a growth control. A well with 80 μl of supplemented Middlebrook 7H9 medium and 20 μl of DMSO alone was also inoculated in each row for refuse anti-mycobacterial effect of DMSO. The plate was incubated at 37°C for 4 weeks. The wells were evaluated after 7, 14, 21 and 28 days and they were compared with the control wells. MIC was defined as the lowest essential oils concentration that exhibited no growth through visual inspection (17).

## RESULTS

**Fig. 1.**
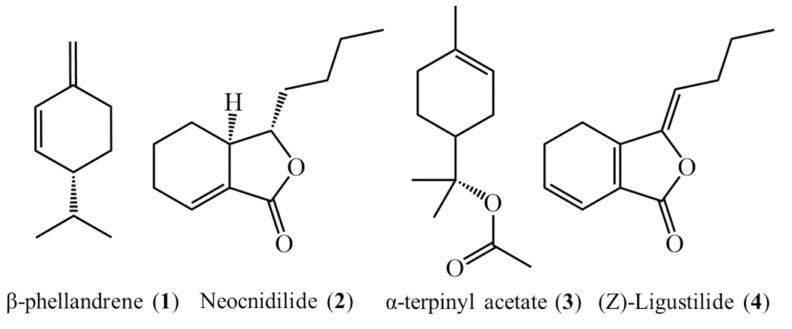
The structure of major compounds in the essential oil

The essential oil was obtained by hydro distillation method on aerial parts of *L. officinale* and the product had yellow-white color with unique odor. Also the yield (v/w%) of essential oil was equal to 2.8%. Identified compounds of *L. officinale* essential oil have been shown in Table 1. The predominate compounds were α-terpinenyl acetate (52.85%), β-phellandrene (10.26%), neocnidilide (10.12%) and (Z)-ligustilide (6.22%), (Fig. 1).

**Table 1.** The chemical composition of the volatile oil from *L. officinale*

**No**	**Compounds**	**RI**	**Percentage**	**Identification**
1	α-pinene	933	0.22	MS, RI
2	sabinene	972	0.13	MS, RI
3	β-pinene	977	5.10	MS, RI
4	p-cymene	1023	0.75	MS, RI
5	β- phellandrene	1029	10.26	MS, RI
6	(Z)-β-ocimene	1036	3.99	MS, RI
7	terpinolene	1087	1.24	MS, RI
8	(Z)-5-dodecen-7-yne	1231	0.37	MS, RI
9	α-terpinyl acetate	1351	52.85	MS, RI
10	methyleugenol	1404	3.16	MS, RI
11	neocnidilide	1734	10.12	MS, RI
12	(Z)-ligustilide	1743	6.22	MS, RI
	Total		97.41	

Due to necessary treatment of TB infection, *L. officinale* essential oil was tested against MDR-TB. Results showed that *L. officinale* essential oil was relatively active on MDR-TB with MIC = 252 μg/ml. In this situation, MIC of isoniazid as pure compound and drug standard was equal to 4 μg/ml. It is notable that, the standard breakpoint value of isoniazid against MTB is equal to 2 μg/ml.

α-Terpinenyl acetate (1), neocnidilide (2), β- phellandrene (3) and (Z) - ligustilide (4) were chosen for molecular docking, because these compounds were specific and major compounds of *L. officinale* essential oil. Isoniazid as the first-line anti-tuberculous drug was selected as standard compound. Isoniazid has shown to act on *M. tuberculosis* by inhibiting a 2-trans-enoyl-acyl carrier protein reductase, called InhA (24). Due to this reason, InhA was selected for doing molecular docking. Docking score of compounds 1, 2, 3 and 4 was equal to −5.5, −6, −5.4, −6.4 kcal/mol respectively, that was comparable with docking score of isoniazid, by −4.6 kcal/mol.

**Fig. 2.**
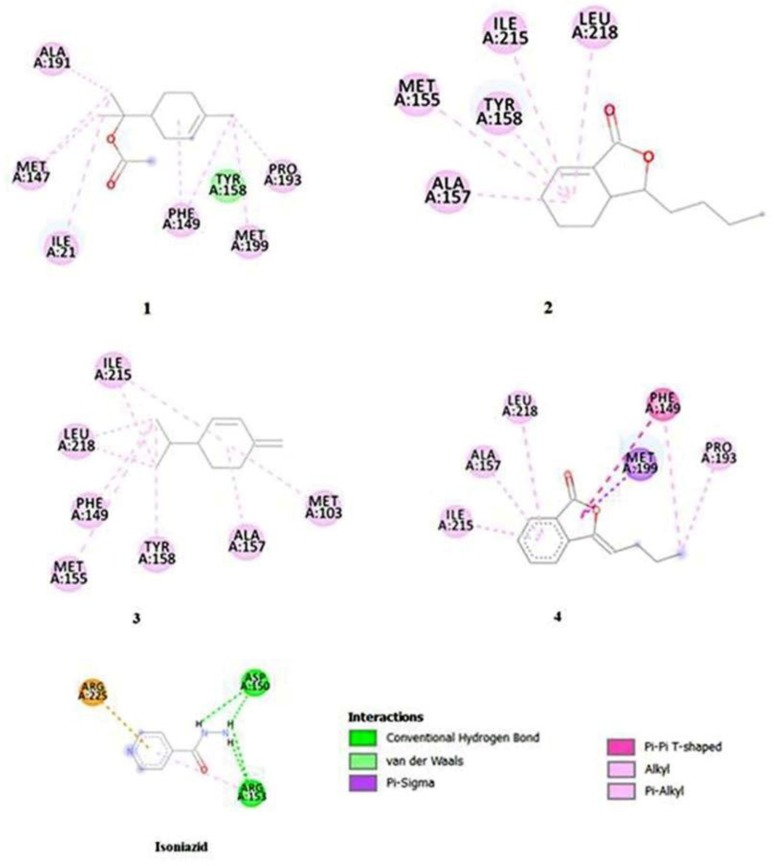
The two-dimensional representation of ligands and receptor (InhA) interaction, the graphs created by aid of by Discover studio package V 4.5

As shown in Fig. 2, compound 1 has alkyl and Pi-alkyl interaction with ALA191, ILE21, PHE149, MET199 and PRO193 of binding site residues of the receptor. There are Pi-sigma, Pi-Pi, alkyl and Pi-alkyl interactions between compound 4 and ILE 215, ALA157, LEU218, PHE149, MET199 and PRO139 as the active site of the receptor. Interactions of compound 2 with receptor include, alkyl and Pi-alkyl with ALA157, MET155, TYR158, ILE215 LEU215 of receptors active site. Compound 3 has alkyl and Pi-alkyl interaction with ILE215, LEU215, PHE149, MET155, TYR158, ALA157 and MET103 of the receptors active site.

## DISCUSSION

In the previous study by Mirjalili et al., antibacterial activity of essential oils from *L. officinale* at different fruiting stages (immature, mature and ripened) of the plant has been studied against *Bacillus subtilis, Staphylococcus epidermidis, S. aureus* and *Escherichia coli*. The mature and ripened fruit oils showed activity with MIC values of 0.9–1.8 mg/ml against *B. subtilis, S. epidermidis.* Also the essential oils at these stages exhibited sensitivity against *E. coli* with MIC=7.2 mg/ml (7).

The composition of *L. officinale* essential oil has been investigated widely and about 190 compounds have been reported. The presence of phthalide compounds is a characteristic for *L. officinale* essential oil. The content of phthalide compounds depends on the geographic location and weather conditions, critical phtalide compounds are propylidenephthalide, (E,Z)-ligustilide, (Z,E)-3-butylidenephthalide and dihydro-butylidenephthalide. Also there are terpenoids compounds including α- and β-phellandrenes, α- and β-pinenes, α-terpinyl acetate and α-terpineol (18). The principle compounds which are present in the essential oil extracted from aerial parts of *L. officinale* were β-phellandrenes (42.5%) and α-terpineol (27.9%) that was reported by Rizi et al. (19). In another study, significant compounds in leaves oil of *L. officinale* were β-phellandrene, α-terpinyl acetate and (Z)-ligustilide, and their content values were equal to 11.3%, 55.8% and 17.0% respectively (20).

*M. tuberculosis* as an intracellular pathogen causes pandemic tuberculosis (TB) (21) leading to the death of about 3 million people per year worldwide. Thus, it is necessary to treat TB through the use of novel drugs, especially in the case of multi-drug-resistant tuberculosis strain. MDR-TB is resistant to conventional drugs such as isoniazid and rifampicin (22–23). Anti-TB activity of *L. officinale* oil is comparable to other results, for example the essential oil of laurel and anise had activity against *M. tuberclosis* with MIC = 100 μg/ml (3). Also the MICs related to three essential oils extracted from *Salvia aratocensis, Turnera diffusa* and *Lippia Americana* were equal to 125 μg/ml against *M. tuberclosis* (4).

α-Terpinenyl acetate, (Z) – ligustilide and neocnidilide as specific and major compounds of *L. officinale* essential oil have more affinity to receptor even more than isoniazid, but isoniazid, unlike these compounds has common hydrogen bond. A conventional hydrogen bond is considered as an essential and vital interaction between drug and receptor. Due to this reason, it is fundamental to make change in the structure of compounds (1, 2 and 3) and investigate SAR and QSAR of these compounds to improve their anti-TB activity.

## CONCLUSION

The *L. officinale* essential oil extracted form aerial parts of this plant was relatively active against MDR *M. tuberculosis*. It will be interesting to isolate compounds of the essential oil and evaluate their anti-TB activity separately, since based on the molecular docking studies done in the present study it has been shown that predominant compounds of the essential oil have had high affinity to inhibit the 2-trans-enoylacyl carrier protein reductase (InhA) as an important enzyme in *M. tuberculosis* cell wall biosynthesis.

## References

[1] Taiwo MO Adebayo OS. Plant essential oil: an alternative to emerging multidrug resistant pathogens. J Microbiol Exp 2017; 5: 1–6.

[2] Edris AE. Pharmaceutical and therapeutic potentials of essential oils and their individual volatile constituents: a review. Phytother Res 2007; 21: 308–323. 17199238 10.1002/ptr.2072

[3] Sergio AO Fabiola CV Guadalupe NM. Evaluation of antimycobacterium activity of the essential oils of cumin (*Cuminumcyminum*), clove (*Eugenia caryophyllata*), cinnamon (*Cinnamomumverum*), laurel (*Laurusnobilis*) and anis (*Pimpinellaanisu*m) against *Mycobacterium tuberculosis*. Adv Biol Chem 2013; 3: 480–484.

[4] Bueno J Escobar P Martínez JR Leal SM Stashenko EE. Composition of three essential oils, and their mammalian cell toxicity and antimycobacterial activity against drug resistant-tuberculosis and nontuberculous mycobacteria strains. Nat Prod Commun 2011;6: 1743–1748. 22224302

[5] Miran M Esfahani HM Farimani MM Ahmadi AA Ebrahimi SN. Essential oil composition and antibacterial activity of *Levisticum officinale* Koch at different developmental stages. J Essent Oil Bear Pl 2018; 21: 1051–1055.

[6] Duke JA. The Green Pharmacy: new discoveries in herbal remedies for common diseases and conditions from the world's foremost authority on healing herbs. Rodale Press, 1997.

[7] Mirjalili MH Salehi P Sonboli A Hadian J Ebrahimi SN Yousefzadi M. The composition and antibacterial activity of the essential oil of *Levisticum officinale* Koch. Flowers and fruits at different developmental stages. J Serb Chem Soc 2010;75: 1661–1669.

[8] Schinkovitz A Stavri M Gibbons S Bucar F. Antimycobacterial polyacetylenes from *Levisticum officinale*. Phytother Res 2008; 22: 681–684. 18350523 10.1002/ptr.2408

[9] Guzman JD Evangelopoulos D Gupta A Prieto JM Gibbons S Bhakta S. Antimycobacterials from lovage root (*Ligusticum officinale* Koch). Phytother Res 2013;27: 993–998. 22899555 10.1002/ptr.4823

[10] Adams RP. Identification of essential oil components by gas chromatography/mass spectorscopy. Allured Publishing Corporation, 2007.

[11] McLafferty FW. Wiley Registry of Mass Spectral Data. Upgrade. Wiley, 2009.

[12] Trott O Olson AJ. AutoDock Vina: improving the speed and accuracy of docking with a new scoring function, efficient optimization and multithreading. J Comput Chem 2010;31:455–461. 19499576 10.1002/jcc.21334 PMC3041641

[13] Jamil AS Mohammad TA Sanjib S Shihab H Alexander G Véronique S. Molecular docking studies on InhA, MabA and PanK enzymes from *Mycobacterium tuberculosis* of ellagic acid derivatives from *Ludwigia adscendens* and *Trewianudiflora*. In Silico Pharmacol 2015;3(1):10. 26820895 10.1186/s40203-015-0014-1 PMC4671986

[14] Zakerbostanabad S Titov LP Bahrmand AR. Frequency and molecular characterization of isoniazid resistance in katG region of MDR isolates from tuberculosis patients in southern endemic border of Iran. Infect Genet Evol 2008;8:15–19. 17988957 10.1016/j.meegid.2007.09.002

[15] Nasiri MJ Rezaei F Zamani S Darban-Sarokhalil D Fooladi AA Shojaei H Drug resistance pattern of *Mycobacterium tuberculosis* isolates from patients of five provinces of Iran. Asian Pac J Trop Med 2014;7:193–196. 24507638 10.1016/S1995-7645(14)60019-5

[16] Askun T Tekwu EM Satil F Modanlioglu S Aydeniz H. Preliminary antimycobacterial study on selected Turkish plants (Lamiaceae) against *Mycobacterium tuberculosis* and search for some phenolic constituents. BMC Complement Altern Med 2013;13:365. 24359458 10.1186/1472-6882-13-365 PMC3878028

[17] Coban AY Birinci A Ekinci B Durupinar B. Drug susceptibility testing of *Mycobacterium tuberculosis* by the broth microdilution method with 7H9 broth. Mem Inst Oswaldo Cruz 2004;99: 111–113. 15057358 10.1590/s0074-02762004000100020

[18] Mirjalili MH Javanmardi J. Handbook of herbs and spices. Abington: Peter KV, Ed. Woodhead Publishing Ltd, 2006.

[19] Riz V Hadjiakhoondi A. The essential oil composition of *Levisticum officinalis* from Iran. Asian J Biochem 2007;2:161–163.

[20] Raal A Arak E Orav A Kailas T Müürisepp M. Composition of the essential Oil of *Levisticum officinale* Koch from some european countries. J Essent Oil Res 2008;20:318–322.

[21] Kardan YJ Haeili M Gizaw Feyisa S Kazemian H Hashemi A Shahraki F Evaluation of efflux pump gene expression among drug susceptible and drug resistant strains of *Mycobacterium tuberculosis* from Iran. Infect Genet Evol 2015;36:23–26. 26325681 10.1016/j.meegid.2015.08.036

[22] Ma C Case R.J Wang Y Zhang HJ Tan GT Hung NV Anti-Tuberculosis constituents from the Stem Bark of *Micromelum hirsutum*. Planta Med 2005;71:261–267. 15770548 10.1055/s-2005-837826 PMC2940840

[23] Macabeo APG Vidar WS Chen X Decker M Heilmann J Wan B *Mycobacterium tuberculosis* and cholinesterase inhibitors from *Voacangaglobosa*. Eur J Med Chem 2011;46: 3118–3123. 21546135 10.1016/j.ejmech.2011.04.025

[24] Marrakchi H Laneelle G Quemard A. InhA, a target of the antituberculous drug isoniazid, is involved in a mycobacterial fatty acid elongation system, FAS-II. Microbiology 2000; 146:289–296. 10708367 10.1099/00221287-146-2-289

